# Spinal bone metastases in gynecologic malignancies: a retrospective analysis of stability, prognostic factors and survival

**DOI:** 10.1186/1748-717X-9-194

**Published:** 2014-09-03

**Authors:** Robert Foerster, Daniel Habermehl, Thomas Bruckner, Tilman Bostel, Ingmar Schlampp, Thomas Welzel, Juergen Debus, Harald Rief

**Affiliations:** Department of Radiation Oncology, University Hospital Heidelberg, Im Neuenheimer Feld 400, 69120 Heidelberg, Germany; Department of Medical Biometry, University Hospital Heidelberg, Heidelberg, Germany

**Keywords:** Bone metastases, Spine, Gynecologic malignancies, Stability, Radiotherapy

## Abstract

**Background:**

The aim of this retrospective study was to evaluate the stability of spinal metastases in gynecologic cancer patients (pts) on the basis of a validated scoring system after radiotherapy (RT), to define prognostic factors for stability and to calculate survival.

**Methods:**

Fourty-four women with gynecologic malignancies and spinal bone metastases were treated at our department between January 2000 and January 2012. Out of those 34 were assessed regarding stability using the Taneichi score before, 3 and 6 months after RT. Additionally prognostic factors for stability, overall survival, and bone survival (time between first day of RT of bone metastases and death from any cause) were calculated.

**Results:**

Before RT 47% of pts were unstable and 6 months after RT 85% of pts were stable. Karnofsky performance status (KPS) >70% (p = 0.037) and no chemotherapy (ChT) (p = 0.046) prior to RT were significantly predictive for response. 5-year overall survival was 69% and 1-year bone survival was 73%.

**Conclusions:**

RT is capable of improving stability of osteolytic spinal metastases from gynecologic cancer by facilitating re-ossification in survivors. KPS may be a predictor for response. Pts who received ChT prior to RT may require additional bone supportive treatment to overcome bone remodeling imbalance. Survival in women with bone metastases from gynecologic cancer remains poor.

## Introduction

Bone metastases are a rare occurrence in gynecologic malignancies and in the majority of cases associated with a poor prognosis [1–8]. Patients (pts) are usually treated with a palliative intention to reduce pain and to preserve functionality. Complications of spinal bone metastases may be severe, especially metastatic spinal cord compression or pathological fractures may tremendously impair patients’ quality of life (QoL) [9].

Treatment is usually multimodal and interdisciplinary. One of the main therapy modalities for bone metastases is radiotherapy (RT). Most frequently patients are treated for pain, but existing or impending instability, neurologic symptoms due to spinal cord compression and post-surgical RT are common indications as well [4, 10]. The stability of vertebral bodies affected by bone metastases is an important aspect in clinical practice and for pts’ QoL. On the one hand disability from pathologic fractures is risked if the vertebral column is not sufficiently stabilized, and on the other hand the usually prescribed surgical corsets add a significant immobilization to the already existing pain. However, mobilization and adequate exercises are of high importance for this subgroup of palliative pts regarding QoL [11] and reduction of the time of hospitalization. Recently we reported on 338 pts with lung cancer in which a significant response towards RT in terms of stability of bone metastases was shown [12].

The purpose of this analysis was to evaluate gynecologic cancer pts with spinal bone metastases treated at our department with a special focus on bone stability after RT, on prognostic factors for stability and on survival.

## Methods

Fourty-four women with thoracic or lumbar spinal bone metastases from gynecologic malignancies were treated at the Department of Radiation Oncology at the University Hospital of Heidelberg between January 2000 and January 2012. Pts’ data were collected from the Heidelberg NCT Cancer Register. The diagnosis was based on CT, MRI or bone scintigraphy findings. Bone metastases had to be located in the thoracic or lumbar spine. After 6 months 34 pts were alive and were, therefore, included in the statistical stability analysis; all 44 pts were included in the statistical survival analysis. Preexisting CT scans were reviewed regarding stability of the osteolytic lesions using the Taneichi score [13]. In pts with more than one metastasis per vertebral body, the one with the worst Taneichi score was assessed. Accordingly, osteolytic metastases with subtypes A to C were classified as stable, and subtypes D to F were classified as unstable. Response was defined as a change from unstable to stable after RT at 3 or 6 months. Pts’ performance status was evaluated with the Karnofsky performance status (KPS) [14]. The characteristics of all pts included in this study are summarized in Table 1. Median follow-up was 6.5 years.

**Table 1:** **Patients’ characteristics**

		n	%
Age (years)			
Median (range)	58 (18–85)		
Karnofsky PS			
60%		4	9
70%		14	32
80%		18	41
90%		8	18
Primarius			
Uterus		16	36
Ovary		14	32
Cervix		9	21
Vulva		4	9
Fallopian tube		1	2
Histology			
Ovaries		14	32
Endometrioid		5	36
Mucinous		2	14
Papillary serous		2	14
Clear cell		5	36
Fallopian tubes		1	2
Adenocarcinoma		1	100
Uterus		16	36
Endometrioid		2	13
Papillary serous		4	25
Clear cell		3	18
Leiomyosarcoma		7	44
Cervix		9	21
Squamous cell		9	100
Vulva		4	9
Squamous cell		4	100
Number of bone metastases			
Mean (range)	2.1 (1–7)		
Solitary		23	52
Multiple		21	48
Spine involvment			
Thoracic		15	34
Lumbar		24	55
Thoracic and lumbar		5	11
Distant metastases			
Brain		15	44
Lung		20	59
Liver		10	29
Skin		1	3

*Abbreviation*: *KPS* Karnofsky performance status.

RT was planned as virtual simulation and performed over a dorsal photon field with the energy 6 MV. PTV covered the vertebral body as well as the vertebral body immediately above and below. Median delivered dose was 30 Gy (range 20–40 Gy) in single fractions of 3 Gy (2–4 Gy) (Table 2).

**Table 2:** **Treatment**

Characteristics		n	%
Radiotherapy dose completed (Gy)			
Single dose (median, range)		3	(2–4)
Cumulative dose (median, range)		30	(20–40)
Indication for radiotherapy			
Pain		21	48
Instability		12	27
Neurologic		6	14
Postoperative		5	11
Treatment for primary site			
Chemotherapy	yes	17	39
	no	27	61
Other treatment for bone metastases			
Surgical corset	yes	8	18
	no	36	82
Bisphosphonates	yes	19	43
	no	25	57

Statistical analysis was done using the SAS software version 9.3 (SAS Institute, Cary, NC, USA). A p-value of p < .05 was considered statistically significant (Chi square and Log-rank test). Overall survival was defined as the time between first diagnosis of malignancy until death from any cause, whereas bone survival was considered to be the time between first day of RT of bone metastases until death from any cause. Survival was plotted according to Kaplan and Meier. Bowker’s test and kappa statistics were calculated to evaluate distribution of the Taneichi score over time. Univariate logistic regression analysis was performed to evaluate possible predictors for stability after 6 months.

## Results

**Figure 1:**
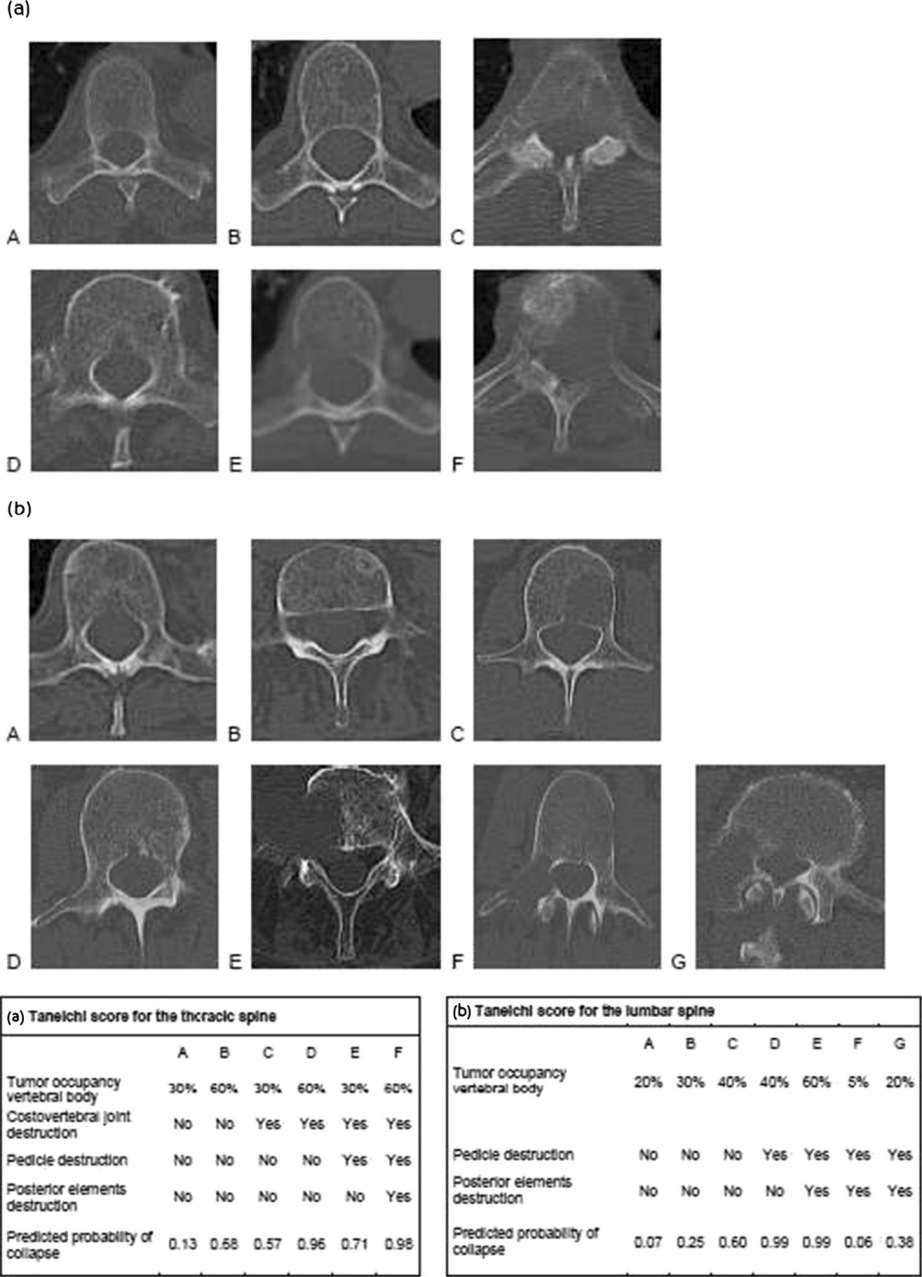
**Taneichi Score: (a) Taneichi Score of the thoracic spine, (b) Taneichi score of the lumbar spine.**

After 6 months 34 pts were alive and were assessed according to the Taneichi score prior to RT, 3 months and 6 months after RT based on CT imaging.Bone metastases were located in the thoracic spine in 34% (n = 15), in the thoracic and lumbar spine in 11% (n = 5) and in the lumbar spine in 55% (n = 24) of the pts. Most frequent subtype according to Taneichi was D (27%; n = 9) (Figure 1). Mean number of spinal metastases per patient was 2 (range 1–7). No pathological fractures occurred.

Sixteen (47%) women had unstable and 18 (53%) pts had stable bone metastases before RT. After 3 months, 62% (n = 21) of metastases were classified as stable and 85% (n = 29) after 6 months (Table 3). No change from stable to unstable was observed. Taneichi subtypes improved in 44% (n = 15) and showed no change in 56% (n = 19) after 6 months. Asymmetry was apparent and correlation was good (p < .001; kappa = .614) (Table 4).

**Table 3:** **The results of Taneichi score evaluation**

	n	%
Stabilitiy before RT		
Unstable	16	47
Stable	18	53
Stability after 3 months		
Unstable	13	38
Stable	21	62
Stability after 6 months		
Unstable	5	15
Stable	29	85

**Table 4:** **Test of symmetry for Taneichi-Score**

		Subtypes 6 months after radiotherapy						
		A	B	C	D	E	F	Total
Subtypes before radiotherapy	**A**	**8**	0	0	0	0	0	8
	**B**	0	**2**	0	0	0	0	2
	**C**	1	2	**5**	0	0	0	8
	**D**	0	2	6	**1**	0	0	9
	**E**	0	0	1	1	**1**	0	3
	**F**	0	0	2	0	0	**2**	4
	**Total**	9	6	14	2	1	2	**34**

This Bowker Test showed the distribution of subtypes of Taneichi-Score before and 6 month after radiation therapy. Asymmetry was apparent (p < 0.001) and the correlation (kappa = 0.614) was good. The evaluation of the distribution of subtypes A to F showed a major change in the direction of improvement over the course of time. Deterioration occurred in no cases, improvement in 44% (n = 15). No change was seen in 56% (n = 19) of the cases.

**Figure 2:**
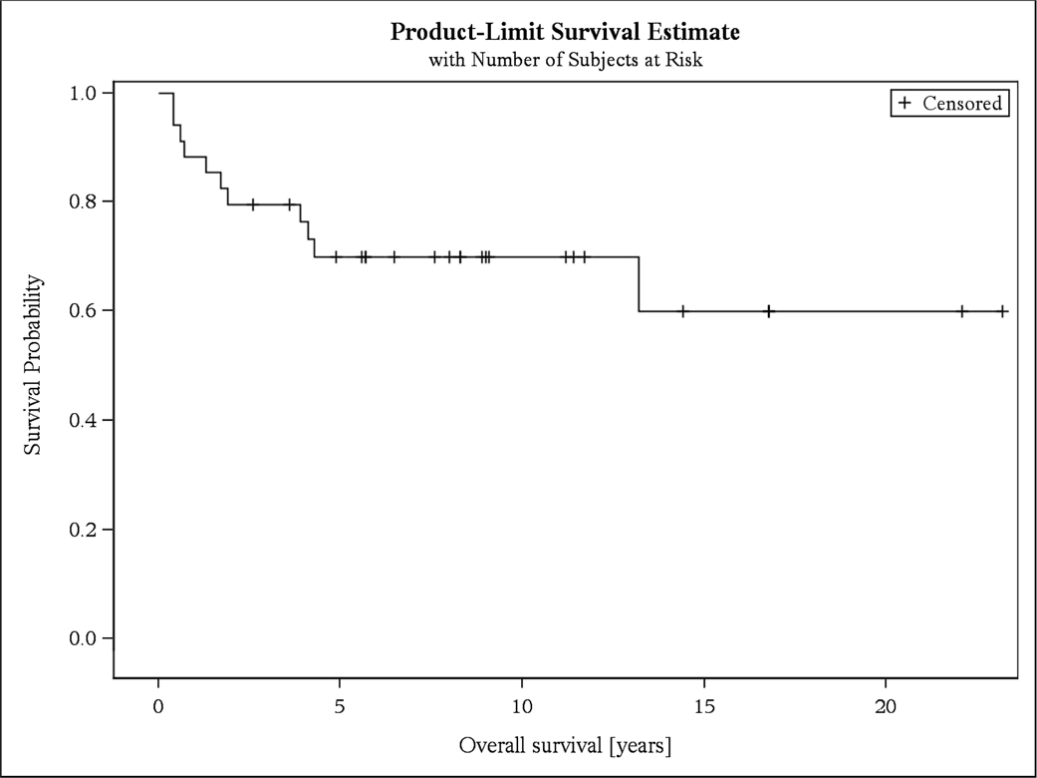
**Overall survival.** Kaplan-Meier curve of overall survival of patients with stable and unstable bone metastases.

**Figure 3:**
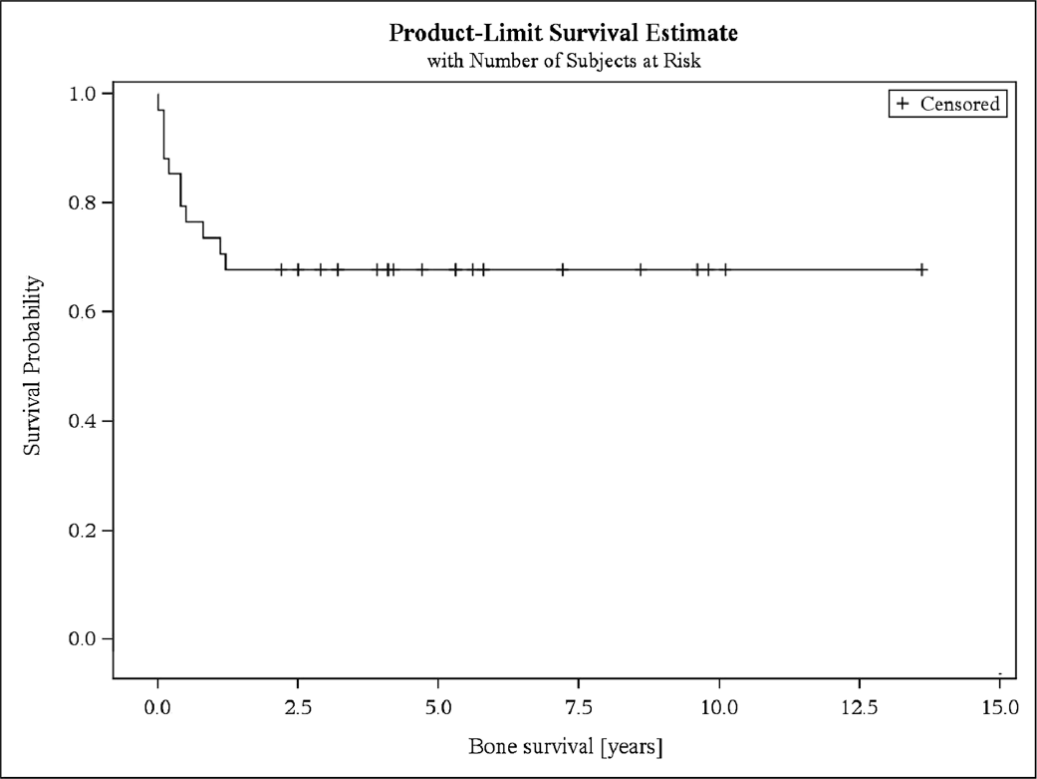
**Bone survival.** Kaplan-Meier curve of bone survival of patients with stable and unstable bone metastases.

KPS >70% prior to RT was significantly correlated with response (p = .037). Additionally pts who did not receive chemotherapy (ChT) prior to RT were significantly more likely to respond (p = .046). Age, prescribed dose, entity of malignancy, location of spinal metastases, number of spinal metastases, bisphosphonate therapy, and use of stabilizing surgical corset were not predictive for response (Table 5).Fourteen pts (32%) died during follow-up, resulting in an overall survival of 69% after 5 years and a bone survival of 73% after 1 year (Figures 2 and 3).

**Table 5:** **Response to radiotherapy after 6 months**

	Non response		Response		p-value
	n	%	n	%	
Primary malignancy					0.794
Uterus	9	75	3	25	
Ovary	6	55	5	45	
Cervix	5	71	2	29	
Vulva	2	67	1	33	
Fallopian tube	1	100	0	0	
KPS					0.037
≤70%	10	71	4	29	
> 70%	7	40	13	60	
Chemotherapy prior to RT					0.046
Yes	12	86	2	14	
No	11	55	9	45	
Location of spinal metastases					0.279
Thoracic	9	75	3	25	
Thoracic and lumbar	3	100	0	0	
Lumbar	11	58	8	42	
Bisphosphonates during RT					0.914
Yes	10	67	5	33	
No	13	68	6	32	
Surgical corset					0.523
Yes	4	80	1	20	
No	19	65	10	35

## Discussion

Bone metastases are comparatively rare in gynecologic malignancies [1–5]. Pts are limited in their quality of life by severe pain and physicians concern about pathological fractures and neurologic consequences such as paraplegia. Stability of spinal metastases is a frequently raised clinical concern in this context and the Taneichi score is an established tool for classification of spinal metastases regarding risk of pathologic fracture or bone instability [13]. In the thoracic spine risk factors are tumor size, and degree of costovertebral joint destruction [15]. In the lumbar part of the spine tumor size and degree of pedicle destruction are the main concern [13]. In our cohort almost half of the patients had unstable metastases at diagnosis. Palliative RT constitutes a potent therapeutic modality for treatment of pain and providing re-ossification [12].

Stability outcome of RT in spinal metastases from gynecologic cancer is still unknown and in previous studies on spinal metastases therapeutic response was only measured in terms of pain control. We were able to demonstrate that RT is capable of improving stability due to re-ossification of osteolytic lesions; only 15% of spinal bone metastases in our cohort of women with gynecologic malignancies remained unstable 6 months after application of RT. The stability before RT was only 53%; whereas 85% were stable after RT.

ChT prior to RT was significantly associated with non-response in our analysis (p = .046). Chemotherapeutics may lead to imbalanced bone remodeling and can cause osteoporosis which in term may prevent response after RT [16]. However, we cannot rule out coincidence because of the small number of pts in our analysis; especially since this is contradictive to the findings of our recent larger analysis of pts with lung cancer [12]. KPS >70% was significantly associated with response to RT (p = .037) which may be explained by continued physical strain to the bones in mobile pts [17, 18].

Overall survival and bone survival were poor and coincide with results from the literature. The longest reported overall survival and bone survival in the literature were 46 months and 25 months respectively [1–8].

## Conclusion

RT is an effective palliative treatment of spinal bone metastases and is capable of improving stability in pts with gynecologic malignancies. KPS may be a predictor for positive response to RT. Pts who underwent ChT prior to RT may require additional bone supportive treatment (bisphosphonates, denosumab, calcium and vitamin D) to overcome bone remodeling imbalance. Survival in pts with bone metastases remains low.

## References

[1] Sehouli J, Olschewski J, Schotters V, Fotopoulou C, Pietzner K (2013). Prognostic role of early versus late onset of bone metastasis in patients with carcinoma of the ovary, peritoneum and fallopian tube. Ann Oncol.

[2] Yoon A, Choi CH, Kim TH, Choi JK, Park JY, Lee YY, Kim TJ, Lee JW, Bae DS, Kim BG (2014). Bone metastasis in primary endometrial carcinoma: features, outcomes, and predictors. Int J Gynecol Cancer.

[3] Yoon A, Choi CH, Kim HJ, Park JY, Lee YY, Kim TJ, Lee JW, Bae DS, Kim BG (2013). Contributing factors for bone metastasis in uterine cervical cancer. Int J Gynecol Cancer.

[4] Thanapprapasr D, Nartthanarung A, Likittanasombut P, Na Ayudhya NI, Charakorn C, Udomsubpayakul U, Subhadarbandhu T, Wilailak S (2010). Bone metastasis in cervical cancer patients over a 10-year period. Int J Gynecol Cancer.

[5] Kumar L, Bhargava VL, Rao RC, Rath GK, Kataria SP (1992). Bone metastasis in ovarian cancer. Asia Oceania J Obstet Gynaecol.

[6] Uccella S, Morris JM, Bakkum-Gamez JN, Keeney GL, Podratz KC, Mariani A (2013). Bone metastases in endometrial cancer: report on 19 patients and review of the medical literature. Gynecol Oncol.

[7] Kehoe SM, Zivanovic O, Ferguson SE, Barakat RR, Soslow RA (2010). Clinicopathologic features of bone metastases and outcomes in patients with primary endometrial cancer. Gynecol Oncol.

[8] Nartthanarung A, Thanapprapasr D (2010). Comparison of outcomes for patients with cervical cancer who developed bone metastasis after the primary treatment with concurrent chemoradiation versus radiation therapy alone. Int J Gynecol Cancer.

[9] Rief H, Heinhold RC, Petersen LC, Rieken S, Bruckner T, Moghaddam-Alvandi A, Debus J, Sterzing F (2013). Neurological outcome after emergency radiotherapy in MSCC of patients with non-small cell lung cancer–a prospective trial. Radiat Oncol.

[10] Habermehl D, Haase K, Rieken S, Debus J, Combs SE (2011). Defining the role of palliative radiotherapy in bone metastasis from primary liver cancer: an analysis of survival and treatment efficacy. Tumori.

[11] Rief H, Akbar M, Keller M, Omlor G, Welzel T, Bruckner T, Rieken S, Hafner MF, Schlampp I, Gioules A, Debus J (2014). Quality of life and fatigue of patients with spinal bone metastases under combined treatment with resistance training and radiation therapy- a randomized pilot trial. Radiat Oncol.

[12] Rief H, Bischof M, Bruckner T, Welzel T, Askoxylakis V, Rieken S, Lindel K, Combs S, Debus J (2013). The stability of osseous metastases of the spine in lung cancer–a retrospective analysis of 338 cases. Radiat Oncol.

[13] Taneichi H, Kaneda K, Takeda N, Abumi K, Satoh S (1997). Risk factors and probability of vertebral body collapse in metastases of the thoracic and lumbar spine. Spine (Phila Pa 1976).

[14] Karnofsky DA, Burchenal JH, MacLeod CM (1949). The Clinical Evaluation of Chemotherapeutic Agents in Cancer. Evaluation of Chemotherapeutic Agents.

[15] Weber MH, Burch S, Buckley J, Schmidt MH, Fehlings MG, Vrionis FD, Fisher CG (2011). Instability and impending instability of the thoracolumbar spine in patients with spinal metastases: a systematic review. Int J Oncol.

[16] Lustberg MB, Reinbolt RE, Shapiro CL (2012). Bone health in adult cancer survivorship. J Clin Oncol.

[17] Ohshima H, Matsumoto T (2012). Space flight/bedrest immobilization and bone. Bone metabolism in space flight and long-duration bed rest. Clin Calcium.

[18] Rief H, Petersen LC, Omlor G, Akbar M, Bruckner T, Rieken S, Haefner MF, Schlampp I, Forster R, Debus J, Welzel T (2014). The effect of resistance training during radiotherapy on spinal bone metastases in cancer patients - a randomized trial. Radiother Oncol.

